# Can ultrasound-guided radiofrequency ablation of genicular nerves of the knee, be performed without locating corresponding arterial pulsations—a cadaveric study

**DOI:** 10.1186/s12891-023-06761-8

**Published:** 2023-08-16

**Authors:** Chinchu Kolakkanni, Nitesh Manohar Gonnade, Ravi Gaur, Ashish Kumar Nayyar, Rambeer Ghuleliya, Abins TK

**Affiliations:** 1 Department of Physical Medicine and Rehabilitation, All India Institute of Medical Sciences, Phase 2 Basni, Jodhpur, Rajasthan India 342005; 2 Department of Anatomy, All India Institute of Medical Sciences, Phase 2 Basni, Jodhpur, Rajasthan India; 3 Department of Physical Medicine and Rehabilitation, Himalayan Institute of Medical Sciences, Swami Ram Nagar, Doiwala, Jolly Grant, Dehradun, Uttarakhand India 248140

**Keywords:** Genicular nerve, Radiofrequency ablation (RFA), Ultrasound, SMGN, SLGN, IMGN, Bony landmarks

## Abstract

**Introduction:**

Given the rising prevalence of knee osteoarthritis, radiofrequency ablation of genicular nerves (RFA) has emerged as a promising treatment option for knee pain. The knee has an extremely complex and variable innervation with nearly 13 genicular nerves described. The frequently ablated genicular nerves are the superomedial (SMGN), the superolateral (SLGN), and the inferomedial (IMGN) genicular nerves. Conventionally, under ultrasound guidance, these nerves are ablated near the corresponding arterial pulsations, but due to the rich vascular anastomosis around the knee joint, identifying the arteries corresponding to these constant genicular nerves can be tedious unless guided by some bony landmarks. In this study, we have evaluated whether it is possible to accurately target these three genicular nerves by just locating bony landmarks under ultrasound in human cadaveric knee specimens.

**Methods:**

Fifteen formalin-fixed cadaveric knee specimens were studied. SMGN was targeted 1 cm anterior to the adductor tubercle in the axial view. For SLGN, in the coronal view, the junction of the lateral femoral condyle and shaft was identified, and at the same level in the axial view, the crest between the lateral and posterior femoral cortex was targeted. For IMGN in the coronal view, the midpoint between the most prominent part of the medial tibial condyle and the insertion of the deep fibers of the medial collateral ligament was marked. The medial end of the medial tibial cortex was then targeted at the same level in the axial view. The needle was inserted from anterior to posterior, with an in-plane approach for all nerves. Eosin, 2% W/V, in 0.1 ml was injected. Microdissection was done while keeping the needle in situ. Staining of the nerve was considered a positive outcome, and the percentage was calculated. The nerve-to-needle distance was measured, and the mean with an interquartile range was calculated.

**Result:**

The accuracies of ultrasound-guided bony landmarks of SMGN, SLGN, and IMGN were 100% in terms of staining, with average nerve-to-needle distances of 1.67, 3.2, and 1.8 mm respectively.

**Conclusion:**

It is with 100% accuracy, that we can perform RFA of SMGN, SLGN, and IMGN under ultrasound guidance, by locating the aforementioned bony landmarks.

**Supplementary Information:**

The online version contains supplementary material available at 10.1186/s12891-023-06761-8.

## Introduction

Choi et al. first described the radiofrequency ablation (RFA) of genicular nerves for knee pain in osteoarthritis patients in 2011, under fluoroscopic guidance. They targeted the superomedial genicular nerve (SMGN), the superolateral genicular nerve (SLGN), and the inferomedial genicular nerve (IMGN) [1]. The genicular nerve RFA was effective in alleviating chronic osteoarthritis (OA) knee pain, in these randomized trials [2–4], longitudinal cohort studies [5–7], and meta-analysis [8–11]. Long-term effects—up to 6 months [7–9, 12] and 1 year [5, 7, 13]- can be obtained if the nerves are accurately targeted.

Genicular nerve RFA is a conditionally recommended method in the ACR 2019 guidelines for the management of OA knee [14]. In OA knee patients with grades 2–4 of the Kellegren-Lawrence (K-L) classification, significant pain Visual analogue scale (VAS) > 5, not responding to simple conservative management [5, 15, 16],—unwilling for total knee arthroplasty (TKA) [17], not fit for TKA [18], along with TKA [19], and even after TKA- genicular nerve RFA is practiced [4, 19–22]. Apart from OA knee patients, cooled RFA was also used for subchondral insufficiency fracture of the knee [23], rheumatoid arthritis (RA) [24], post-Anterior cruciate ligament (ACL) reconstruction [25], and juvenile idiopathic arthritis (JIA) patients with chronic knee pain [26].

The factors that determine the effect of RFA are disease severity, genicular nerve course, nerve needle proximity, and the Gauge (G) of the RFA needle used. The size of the lesion produced by an RFA needle is proportional to its electrode width [27, 28]. As a practical rule, the nerve has to be within twice the electrode's width. For adequate nerve lesions, using smaller electrodes of 20 G or 22 G, the electrode should be on the nerve, and a change of 1 mm can miss the nerve. But with larger electrodes of 18 and 16 G, there can be more flexibility [29]. Hence, the nerves should be precisely localized for ablation.

Out of 13 genicular nerves described around the knee [30], the most targeted nerves are the SMGN, SLGN, and IMGN. It is well established that there are five constant genicular nerves innervating the anterior knee capsule: SMGN, SLGN, IMGN, the recurrent genicular nerve, and the infrapatellar branch of the saphenous nerve (IPBSN) [30, 31]. But the SMGN, SLGN, and IMGN are frequently ablated, and hence included here.

### Superomedial genicular nerve

SMGN was previously described as a branch from the tibial nerve, branching from the upper part of the popliteal fossa and curling around the femur, and making bony contact at the shaft and the condyle junction [1, 15, 32–34] where it can be targeted. But with further studies, this nerve was found to be a terminal branch of the nerve to the vastus medialis (NVM), which descends with the adductor magnus tendon and makes bony contact around 1 cm anterior to the most prominent part of the adductor tubercle. There are different approaches prevailing for targeting this nerve. Conventionally, the bony cortex near the corresponding artery at the shaft and medial epicondyle junction is used as the landmark for targeting this nerve [24, 35–39]. The junction of the shaft and medial epicondyle of the femur in the coronal plane and half of the depth of the femur in the axial view is also used [40]. Another approach is to target in relation to the adductor tubercle [30, 31, 41–43], which is nearly 1 cm anterior to the adductor tubercle [33].

### Superolateral genicular nerve

Previously, SLGN was described as a branch from the vastus lateralis [41, 42, 44]. Further anatomical studies revealed that SLGN originated from the sciatic nerve in 90% of the studied specimens and the common peroneal nerve in the remaining 10%. [30, 45] Even though there is variation in origin, the distal course is constant [41]. Conventionally, the nerve is targeted at the bony cortex, near the corresponding artery, over the junction of the shaft and lateral femoral epicondyle [24, 35–39, 42, 44]. Recently described techniques include, targeting the superolateral popliteal fossa, medial to biceps femoris tendon, around 2.6 cm proximal to the most prominent part of the lateral femoral epicondyle visualized under ultrasound [46], or locating the junction of the shaft and lateral epicondyle of the femur in the coronal plane and to target either half of the depth of femur in axial view [32, 40] or the crest separating lateral and posterior cortex, in axial view [43].

### Inferomedial genicular nerve

The IMGN was described as arising from the tibial nerve in the popliteal fossa and curving around the tibia to emerge anteriorly, deep to the medial collateral ligament, at the midpoint between the most prominent part of the medial tibial condyle and the medial collateral ligament's (MCL) initial insertion [1, 30, 32–34, 41, 47]. Conventionally, it is targeted at the junction of the medial tibial condyle and shaft in the coronal plane and half the girth of the tibia in the axial plane [32, 43, 48] and near the corresponding artery [24, 35–40]. Another technique is to target half the distance between the most prominent part of the medial tibial condyle and the insertion of deep fibers of the medial collateral ligament visualized under ultrasound [33, 34].

Though there have been detailed cadaveric study to describe newer anatomical targets, to ablate these genicular nerves in 2021 [43], still genicular nerve ablation is performed with conventional techniques, as evidenced by the studies that succeeded [35, 38]. This may be due to the lack of evidence from multiple studies. So, studies on a greater number of populations from different ethnicities can augment the level of evidence for the same. Hence this study was done to evaluate the accuracy of genicular nerve block, by ultrasound-guided identification of the bony landmarks, described below.

## Methodology and techniques

A total of 15 formalin-fixed human cadaveric knee specimens were studied. The age, gender, and length of the cadavers were documented. Ultrasound scanning was performed using the 5–13 MHz linear probe of the Venue Go R3 ultrasonography (USG) machine.

**Fig. 1:**
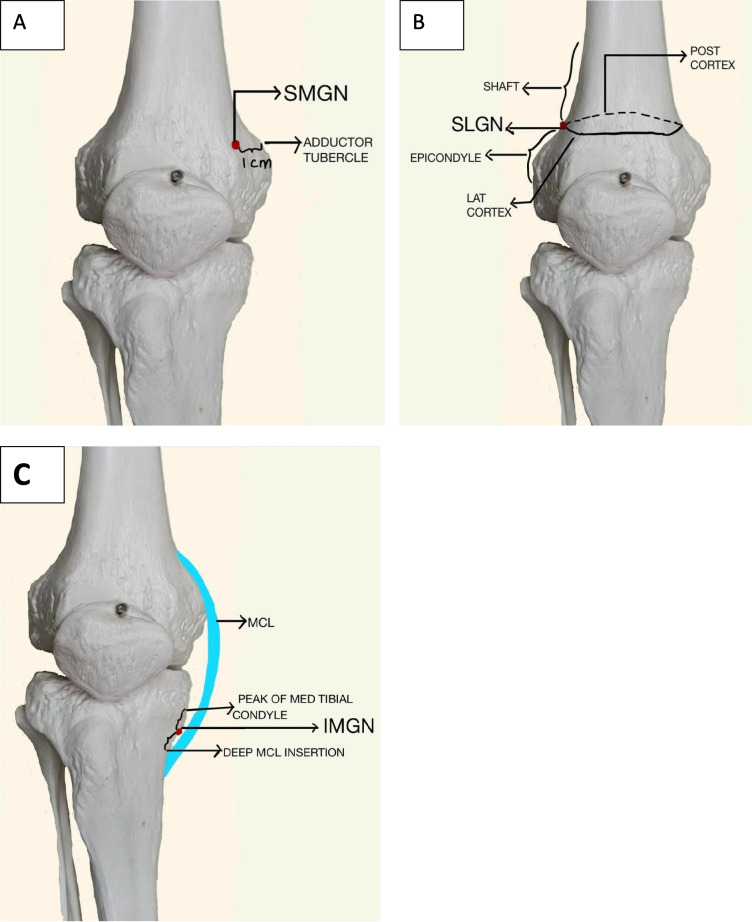
Schematic representation of target points **A**-SMGN, **B**-SLGN, **C**-IMGN LAT: lateral, MED: medial, POST: posterior, MCL: medial collateral ligament

**Fig. 2:**
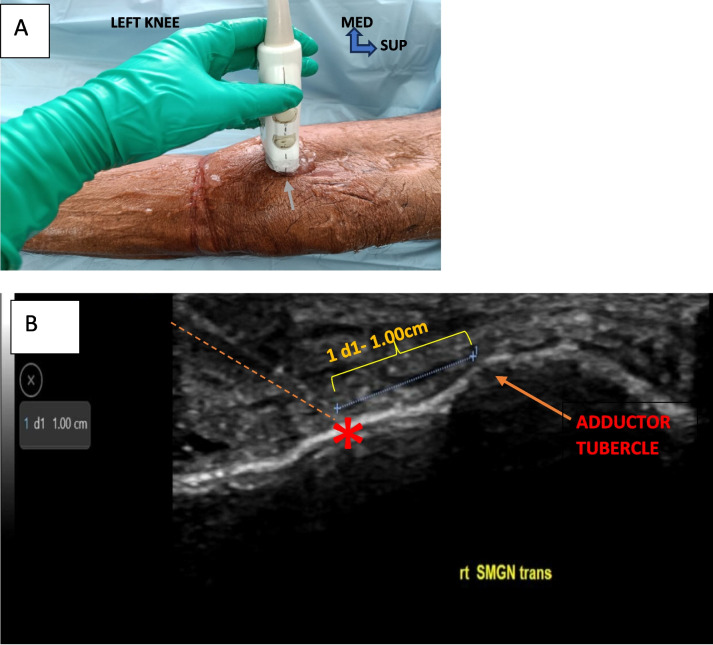
SMGN identification under USG guidance. **A** Probe position. The needle entry is shown by a grey arrow. **B** The transverse view at the level of the adductor tubercle. The target point of SMGN is marked with a red Asterix. The needle tract is marked by an orange dotted line. Rt- right. Trans- Transverse view

To locate the SMGN landmark (Fig. 1A) the probe was placed in the coronal plane, visualizing the medial joint line, and it was translated superiorly to identify the adductor tubercle. At this point, the probe orientation was changed to a transverse direction, and one cm anterior to the adductor tubercle was measured and identified (Fig. 2).

**Fig. 3:**
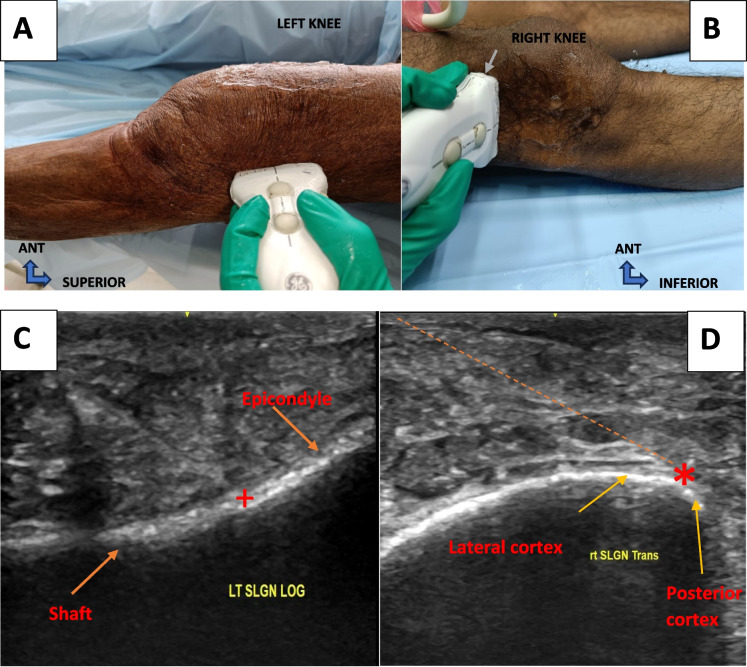
SLGN identification under USG guidance. **A- **probe position for coronal view. **B-** Probe position for transverse view. The needle entry point is shown the by a grey arrow. **C-** longitudinal view. Plus shows the junction of the shaft and lateral femoral epicondyle. **D-** Transverse view showing the target with red Asterix. The needle track is marked by an orange dotted line. LOG: longitudinal, LT: left

For the SLGN landmark (Fig. 1B) the probe was placed in the coronal plane, visualizing the lateral joint line, and superiorly translated to identify the junction of shaft and condyle. Then the probe was rotated by 90 degrees to a transverse view to locate the crest between the lateral and posterior cortex (the lateral most end of the lateral cortex) (Fig. 3).

**Fig. 4:**
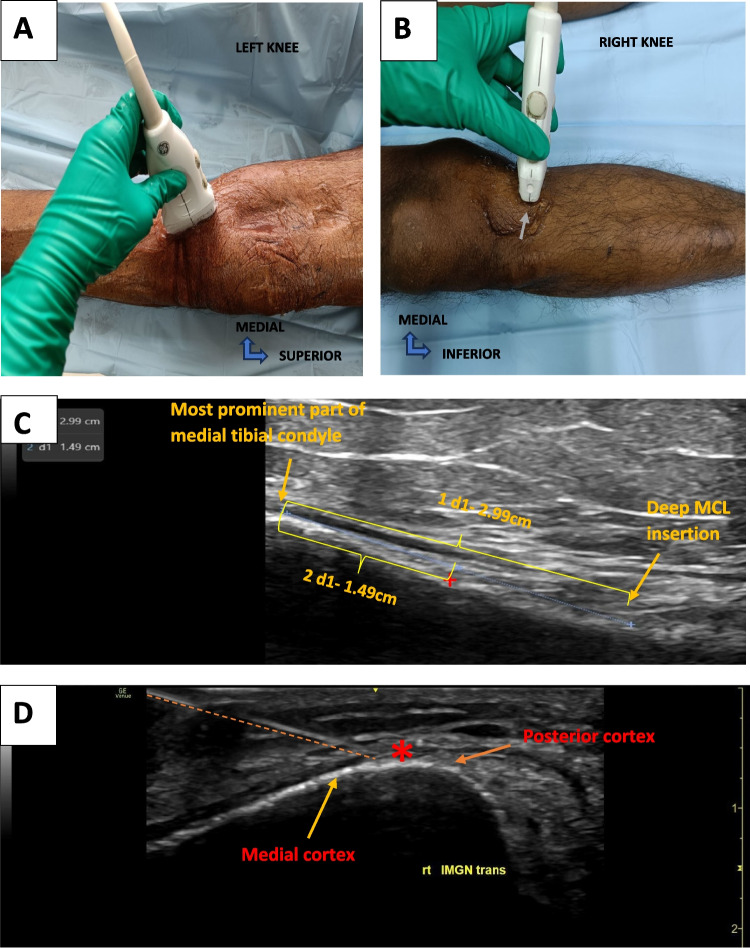
IMGN identification under USG guidance. **A-** Probe position for longitudinal view. **B-** Probe position for transverse view. The needle entry is shown by the grey arrow. **C-** shows the coronal view, plus sign marks the midpoint between the most prominent part of the medial tibial condyle and insertion of deep MCL. **D-** Transverse view, which shows the target with a red Asterix. The needle track is marked by an orange dotted line. Rt- right, trans-transverse view

The IMGN landmark (Fig. 1C), was located with the probe placed in the coronal plane, visualizing the medial joint line, and it was translated inferiorly to visualize the insertion of deep fibres of the MCL. The midpoint between the most prominent part of the medial tibial condyle and the insertion of the medial collateral ligament was identified, and the orientation of the probe was rotated by 90 degrees to a transverse view to locate the medial end of the medial cortex (Fig. 4).

For all three nerves, a 22-gauge Quincke 38-mm spinal needle was inserted, from anterior to posterior with an in-plane approach. 0.1 ml of eosin, 2% W/V was injected. Dissection was done while keeping the needles in situ, under visualization through the SurgiTel EVK450, a loupe microscope, and using microdissection scissors.

### Dissection

A cruciform incision with no: 11 surgical blades centering the needle was made for all three nerves. The subcutaneous tissue and fat were removed with microdissection scissors without altering the needle position. The needles were forcefully pierced into the periosteum and bone for anchorage.

For SMGN, the vastus medialis muscle was cut longitudinally, in the middle of the thigh. The fascial tunnel of the vastus medialis was identified. The SMGN was identified inside this tunnel. The nerve was traced proximally to identify its branching from the nerve to the vastus medialis. Then it was traced distally, carefully dissecting the rest of the tissue under visualization through the Loupes microscope.

To dissect the SLGN, the cadaver was positioned in a side-lying position. After skin and subcutaneous dissection, the biceps femoris muscles, the iliotibial tract, and their fascia were cut through the needle and retracted. In this plane, the fatty tissue was carefully removed under visualization through a loupe microscope, keeping the soft tissue just around the needle for anchorage. The common peroneal nerve was identified. The branches of the nerve were dissected carefully, and SLGN was identified as making bony contact near the epicondyle-shaft junction. Then, the remaining soft tissue around the needle was removed by forcing the needle into the periosteum and holding it.

For IMGN, the cadaver was positioned supine. After removing the skin and subcutaneous tissue in the aforementioned manner, the deep fascia was cut through the needle. The MCL was visible. The MCL was cut transversely through the needle and retracted proximally and distally. The fascia was removed using microdissection forceps under a loupe microscope. The needle was advanced to pierce the periosteum to maintain stability. The IMGN and vessels were identified.

For all the three nerves, nerve-to-needle distance were measured using a vernier callipers of 0.1 mm accuracy and 0.01 mm accuracy.

## Result

Out of the 15 cadaveric knee specimens studied, 10 (67%) were from male cadavers. The median length of the cadaver is 162.0 cm, with an interquartile range of 154.5 to 169.0 cm. The median age of the cadavers was 66, with an interquartile range of 61 to 70 (Table 1).

**Table 1:** Demographic details of the cadavers

Characteristic	*N* = 15^a^
**LENGTH**	162.0 (154.5, 169.0)
**AGE**	66 (61,70)
**SEX**	
F	5 (33%)
M	10 (67%)

^a^*Median (Q1, Q3)*; *n (% frequency)*

### Dissection findings

#### SMGN

**Fig. 5:**
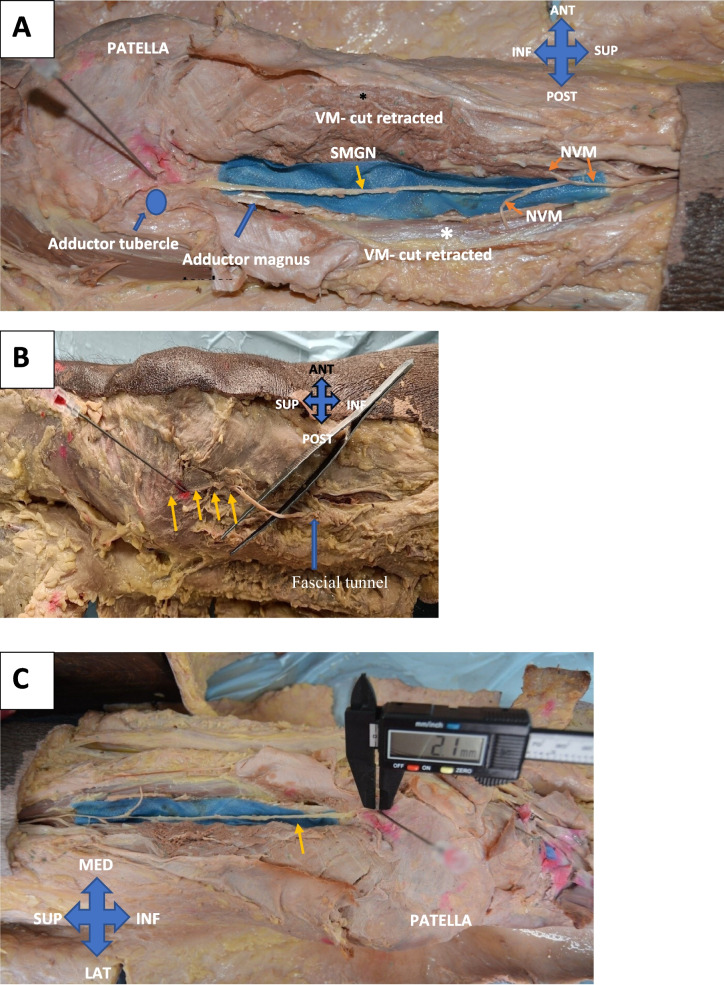
Dissected SMGN. **A-** SMGN originating from the NVM. **B-** The SMGN is partially dissected out to show its passage through the fascial tunnel. **C** The distance from nerve to needle tip measured. VM- vastus medialis (All are right knee specimens)

The SMGN was the distal branch of the NVM in 100% of the specimens (Fig. 5A). The nerve at the middle of the thigh entered a fascial tunnel, and it took an exit from the fascial tunnel near the distal third of the femur (Fig. 5B). After which it descended down along with the adductor magnus tendon. The nerve made contact with the femur, at 10.15 mm (average) anterior to the adductor tubercle and the adductor magnus insertion. Figure 5C shows the measurement of nerve to needle distance.

#### SLGN

**Fig. 6:**
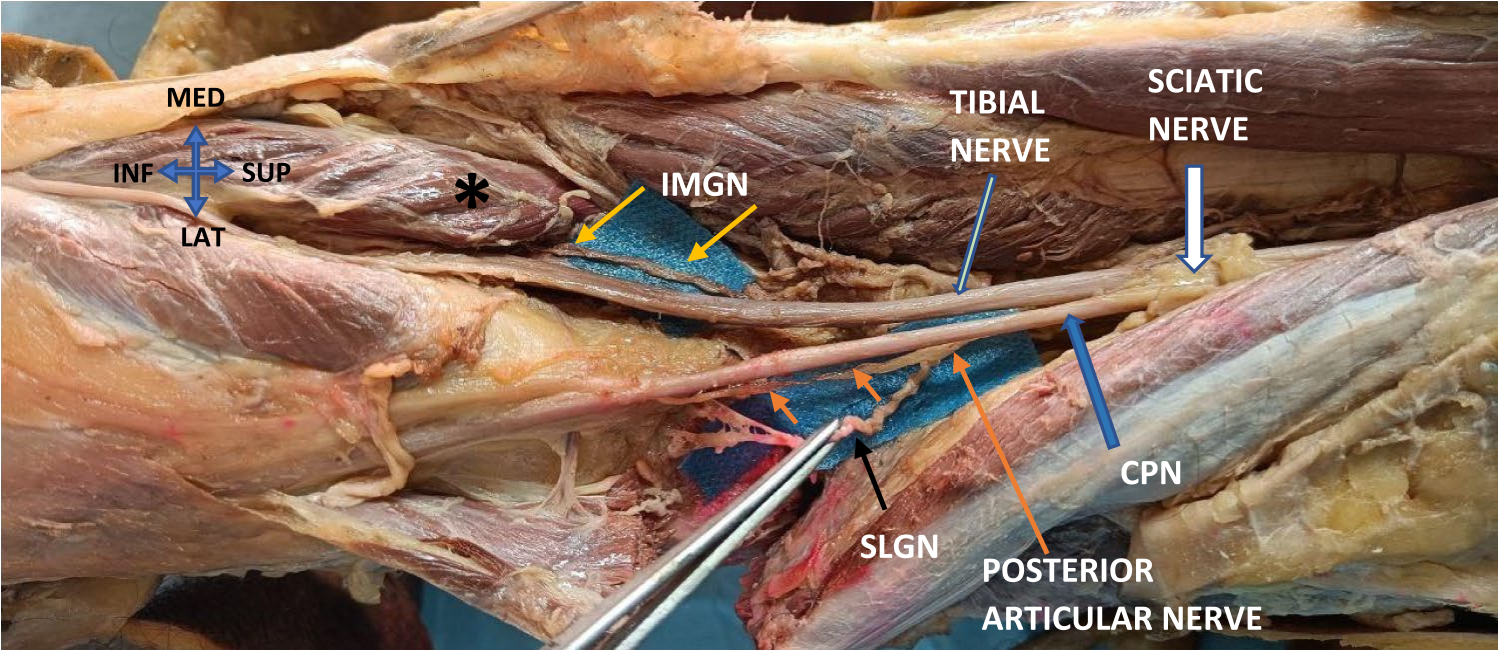
Popliteal fossa dissection- IMGN, and SLGN origin- The IMGN (Yellow arrow) arising from the tibial nerve and passing deep to the medial gastrocnemius (*). The SLGN branches from the posterior articular nerve, which in turn branches from the sciatic nerve

**Fig. 7:**
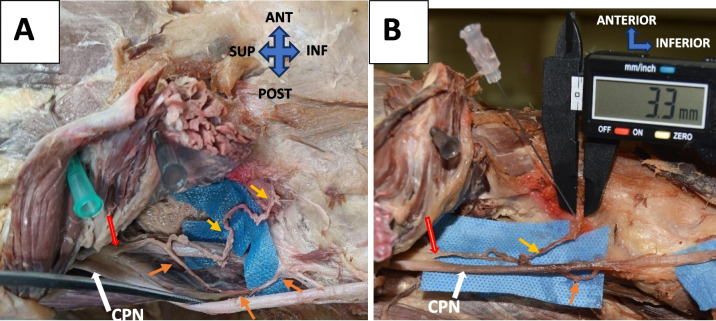
**A** Course of SLGN – Branching from the common peroneal nerve (orange thick arrow), and dividing into 2 branches. Yellow arrow- transverse branch. Orange arrow- descending branch. CPN- Common peroneal nerve (thick white arrow). **B** nerve to needle distance measured. (Both are right knee specimens)

SLGN was originating from the posterior articular nerve (branch of either the sciatic nerve, or the common peroneal nerve) in 73 percent of specimens (*n* = 11) (Fig. 6), or as a direct branch from the common peroneal nerve in 27 percent of specimens (*n* = 4) (Fig. 7A). After making bony contact at the shaft-condyle junction, it divided into two branches. We noticed that, in all specimens, the transverse branch was getting stained as the descending branch already descended towards the lateral femorotibial space. The nerve’s path was extremely tortuous (Fig. 7A). Figure 7B shows the measurement of nerve to needle distance.

#### IMGN

**Fig. 8:**
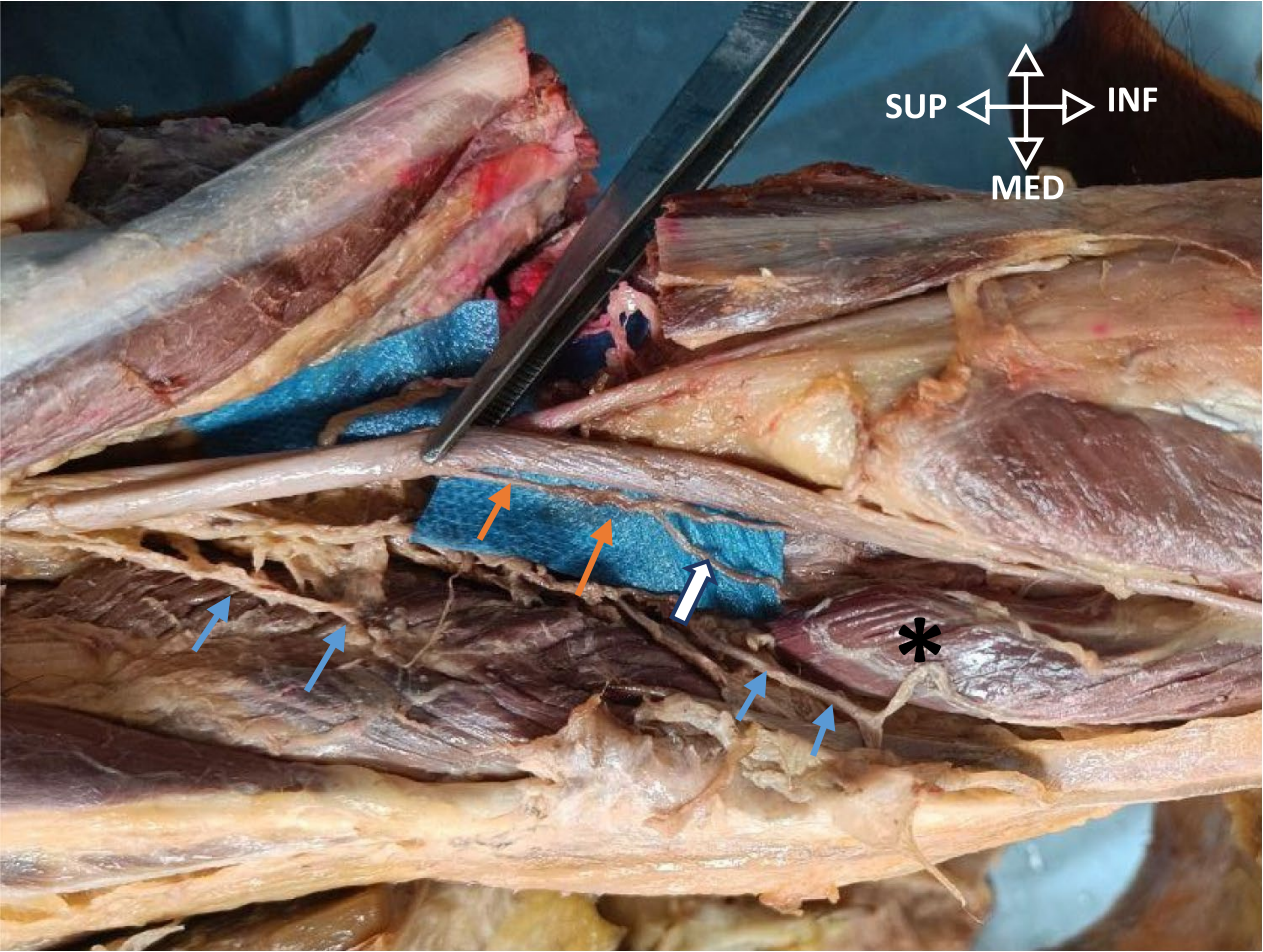
Popliteal fossa dissection- IMGN origin from the articular nerve. Shows the long articular nerve (orange arrow) originating from tibial nerve to supply the posterior capsule, and gives off IMGN (white arrow), which traverses deep to the medial gastrocnemius (*). Other muscular branches are shown in the blue arrow. Right knee specimen

**Fig. 9:**
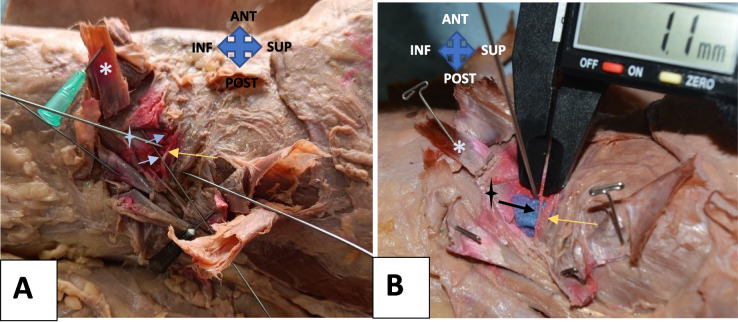
Dissected IMGN. **A** and **B** IMGN marked with arrows. The Asterix shows the superficial MCL. The star shows deep MCL. The yellow arrow shows the inferomedial genicular vessels. B: measurement of IMGN to needle tip. (Both are right knee specimens)

IMGN originated from the tibial nerve in the popliteal fossa, in 40 percent (*n* = 6) of the knee specimens (Fig. 6). In 60 percent (*n* = 9) of the knee specimens, it was found to be branching from the long articular branch of the sciatic or tibial nerve (Fig. 8). Invariable of its origin, it traversed deep to the medial gastrocnemius, curving around the tibia from posterior to anterior as a neurovascular bundle beneath the medial collateral ligament (Fig. 9A). Figure 9B shows the measurement of nerve-to-needle distance.

The staining characteristics and nerve-to-needle distance of the three nerves are shown in Table 2.

**Table 2:** Staining and nerve-to-needle distance of genicular nerves

Characteristic	SMGN (*N* = 15^a^)	SLGN, (*N* = 15^a^)	IMGN, (*N* = 15^a^)
Stained	15 (100%)	15 (100%)	15 (100%)
Needle-to-nerve distance	1.67 (1.49, 1.84)	3.2 (3.1, 3.4)	1.80 (1.07, 2.04)

^a ^*n (% frequency); Median (Q1, Q3)*

## Discussion

The bony landmarks that we have used for genicular nerve localization are found to be highly accurate. Hence, accurate genicular nerve RFA can be done, even if corresponding arterial pulsations are unidentifiable. During the dissection, we also found that the nerve courses were in accordance with the described landmarks.

### SMGN

The SMGN was the distal branch of the NVM in 100% of the specimens, as was described in these anatomical studies [30, 31]. The nerve was stained in 100 percent of the specimens, just like Yasar et al. found in their cadaveric study [33]. Wong et al., Kesikburun et al., Ahmed and Arora, Cankurtaran et al., Gupta et al., and Ghai et al. targeted the nerve anterior to the adductor tubercle in OA knee patients and found to be effective in alleviating pain [34, 36, 42, 44, 46, 49].

The average nerve-to-needle distance measured was 9.9 mm, which is well within any RFA ablation diameter.

### SLGN

SLGN had two different origins, as described by Fonkoue et al. and Kim et al. [30, 45]. Our study landmark was 100 percent accurate in terms of staining, like in the previous study by Fonkoue et al. [43]. Further the average nerve-to-needle distance measured was 3.2 mm, which is within the RFA diameter. Hence, at this point, SLGN can be ablated with high accuracy for RFA.

We also found that the crest between the lateral cortex and posterior cortex described by Founke et al. [43] is anatomically the same as half the girth of the femur described by Vanneste et al. and Mittal et al. [32, 40] which is further same as the lateral most end of the lateral cortex. Hence all these descriptions can be used to locate the nerve in axial view.

### IMGN

IMGN originated from the tibial nerve in the popliteal fossa, in 40 percent (*n* = 6) of the knee specimens, as was described by Tran et al. and Robert et al. [50, 51] (Fig. 6) In 60 percent (*n* = 9) of the knee specimens, it was found to be branching from the long articular branch of the sciatic or tibial nerve, as was described by Kim et al. [45] (Fig. 8). Invariable of its origin, it traversed deep to the medial gastrocnemius, curving around the tibia from posterior to anterior as a neurovascular bundle beneath the medial collateral ligament (Fig. 9A).

In this study, we have targeted the nerve midway between the most prominent part of the medial tibial condyle and the insertion of the deep fibers of MCL, as was done by Yasar et al., Wong et al., Kesikburun et al., and Ghai et al. [33, 34, 46, 52]. We found this to be 100% accurate in in terms of staining. The nerve-to-needle distance was 1.8 mm, and this can be used for RFA.

## Conclusion

Our study results showed that the ultrasound guided RFA of SMGN, SLGN, and IMGN can be done, with 100% accuracy by using the described bony landmarks even if the corresponding arterial pulsations are unidentifiable.

### Limitations

Less number of cadaveric knee specimens was a limitation.

### Supplementary Information

**Additional file 1: Supplementary table 1.** Staining characters and nerve-to-needle distance for USG-guided bony landmark techniques.

## Data Availability

All data given in study is provided in Supplementary table 1 along with the legend.

## Abbreviations

SLGN: Superolateral genicular nerve
IMGN: Inferomedial genicular nerve
W/V: Weight by volume
RFA: Radiofrequency ablation
K-L: Kellegren- Lawrence
VAS: Visual analogue scale
TKA: Total knee arthroplasty
RA: Rheumatoid arthritis
JIA: Juvenile idiopathic arthritis
IPBSN: Infrapatellar branch of saphenous nerve
NVM: Nerve to vastus medialis
MCL: Medial collateral ligament
USG: Ultrasonography
