# Supplementary material for: Can ultrasound-guided radiofrequency ablation of genicular nerves of the knee, be performed without locating corresponding arterial pulsations—a cadaveric study
Source: BMC Musculoskelet Disord. 2023 Aug 16;24:654. doi: 10.1186/s12891-023-06761-8 (PMC10429091; doi:10.1186/s12891-023-06761-8)
Supplement: Supplementary file 1 — Additional file 1: Supplementary table 1. Staining characters and nerve-to-needle distance for USG-guided bony landmark techniques. [file 12891_2023_6761_MOESM1_ESM.docx]

**SUPPLEMENTARY TABLE 1**

**STAINING CHARACTERS AND NERVE-TO-NEEDLE DISTANCE FOR USG-GUIDED BONY LANDMARK TECHNIQUES.**

| **SL NO.** | **HEIGHT** | **AGE** | **SEX** | **SMGN** | | | **IMGN** | | **SLGN** | |
| --- | --- | --- | --- | --- | --- | --- | --- | --- | --- | --- |
|  |  |  |  | **STAIN** | **NND** | **AT-SMGN** | **STAIN** | **NND** | **STAIN** | **NND** |
| 1 | 162 | 70 | M | 1 | \| 2.01 \| \| --- \| \|  \| | 9.69 | 1 | 1.1 | 1 | 3.1 |
| 2 | 162 | 66 | M | 1 | 2.1 | 10.62 | 1 | 1.01 | 1 | 3.3 |
| 3 | 159 | 61 | M | 1 | 1.66 | 11.06 | 1 | 0.98 | 1 | 2.9 |
| 4 | 160 | 59 | M | 1 | 1.34 | 11.4 | 1 | 1.03 | 1 | 3.21 |
| 5 | 156 | 62 | F | 1 | 1.22 | 10.58 | 1 | 1 | 1 | 2.9 |
| 6 | 152 | 71 | F | 1 | \| 9.22 \| \| --- \| \|  \| | 2.87 | 1 | 2.4 | 1 | 3.01 |
| 7 | 174 | 68 | M | 1 | 1.8 | 12.02 | 1 | 2.01 | 1 | 4.23 |
| 8 | 168 | 58 | M | 1 | 1.53 | 10.42 | 1 | 2.08 | 1 | 3.42 |
| 9 | 170 | 60 | M | 1 | 1.67 | 8.56 | 1 | 1.88 | 1 | 4.2 |
| 10 | 170 | 63 | M | 1 | 1.56 | 9.62 | 1 | 2.88 | 1 | 3.26 |
| 11 | 152 | 66 | F | 1 | 1.78 | 13.69 | 1 | 2 | 1 | 4.32 |
| 12 | 168 | 75 | M | 1 | 1.88 | 11.59 | 1 | 2.7 | 1 | 3.45 |
| 13 | 153 | 76 | F | 1 | 1.68 | 8.98 | 1 | 1.8 | 1 | 3.2 |
| 14 | 153 | 80 | F | 1 | 1.43 | 7.98 | 1 | 1.5 | 1 | 3.08 |
| 15 | 170 | 77 | M | 1 | 1.45 | 9.45 | 1 | 1.45 | 1 | 3.14 |

**SUPPLEMENTARY TABLE 1:** The table shows the data from 15 cadaveric knee specimens in which the ultrasound-guided bony landmarks were targeted for SMGN, IMGN, and SLGN. The cadaver number is the unique number given to the cadavers. The height was measured in centimetres. The STAIN denotes staining outcomes (1: stained, 0: not stained). NND is the nerve-to-needle distance (in millimetres) measured using a vernier calliper. AT-SMGN: The distance of SMGN from adductor tubercle. The average distance of SMGN from the adductor tubercle is mentioned in the manuscript, which was calculated from this data. SMGN: Superomedial genicular nerve, IMGN: inferomedial genicular nerve, SLGN: Superolateral genicular nerve. STAIN: Staining outcomes, NND: Nerve to needle distance, AT-SMGN: Adductor tubercle to SMGN distance.
